# Unpacking the Relationship between Fear Motives and Self-Control Strategies among Managers: The Mediating Role of Intrusive Thoughts

**DOI:** 10.3390/bs13050384

**Published:** 2023-05-06

**Authors:** Cafer Bakaç, Hugo M. Kehr

**Affiliations:** TUM School of Management, Technical University of Munich, 80333 Munich, Germany

**Keywords:** implicit fear motives, self-control strategies, intrusive thoughts, positive affect, field study

## Abstract

In motive research, only a handful of studies have appeared on the correlates and antecedents of fear motives. In this research, we contribute to both research and practice by investigating the relationships between fear motives, intrusive thoughts, self-control strategies, and positive affect. We propose that fear motives, similar to trait anxiety, are positively associated with intrusive thoughts and that intrusive thoughts, in return, are negatively related to the frequency with which individuals employ self-control strategies. Finally, we propose that the frequency of self-control strategies is positively associated with positive affect. To test these, two field studies with managers (Study 1: *N* = 100 and Study 2: *N* = 80) were conducted. Bayesian mediation analyses showed that in both Study 1 and Study 2, fear motives were positively associated with intrusive thoughts, and intrusive thoughts were negatively related to self-control strategies. Additionally, in line with predictions, intrusive thoughts mediated the fear motives-self-control strategies relation. Finally, in Study 2, we found self-regulation strategies to be significantly and positively associated with positive affect. The theoretical and practical implications of the study are discussed.

## 1. Introduction

Organizational and personality psychologists have long been interested in implicit motives as covariates of important work and personality variables such as work performance [1], counterproductive work behaviors [2], and goal progress [3]. They are described as nonconscious and stable personality dispositions that orient, select and energize individual behaviors [1,4]. A large body of research has focused on The Big Three motives, including achievement, affiliation, and power [1,2,5,6]. Several researchers have divided The Big Three motives into hope (or approach) and fear (or avoidance) components [4,7]. Individuals with a high hope motive component seek positive goal states and experience positive emotions upon successful goal attainment. By contrast, individuals with a high fear motive component try to avoid negative and undesirable goal states and, with successful avoidance, experience a decrease in their negative emotions [8]. 

Since its inception, researchers have studied the hope component of motives extensively [2,5,9,10]. These researchers found that the hope component is negatively associated with counterproductive work behaviors [2], and positively associated with negotiation initiation [5], success in politics [9], and networking behaviors [10]. Conversely, studies on the fear component of motives (henceforth fear motives), have been scant. From this research, there is evidence that individuals who have high fear motives have higher levels of neuroticism [11], put more time into practicing sports [12], experience negative moods, and reduced goal commitment when they daydream about their goal attainment [13]. However, more research is needed to unravel the further correlates, mechanisms, and antecedents of fear motives.

In this study, we aim to extend the literature on fear motives by investigating the relationships between fear motives, intrusive thoughts, self-control strategies, and positive affect. Building on the previous literature, we conceive fear motives as a unified construct [14], and integrating the previous literature, we claim that similar to trait anxiety, fear motives interfere with inhibition function [15,16], which results in difficulty in suppressing intrusive thoughts [17]. Furthermore, the effort individuals put into suppressing these intrusive thoughts depletes attentional resources, which in turn, impairs performance [18]. Based on this, we also claim that intrusive thoughts deplete resources, which makes it difficult for individuals to call for further self-control strategies. 

With this research, we contribute to the literature in several ways. First, by employing fear motives as antecedents of intrusive thoughts, self-control strategies, and positive affect, we extend the literature on the correlates and antecedents of fear motives. Second, we examine possible mechanisms through which fear motives are associated with individuals’ frequency with which they employ self-regulation strategies [19]. In order to ensure the applied relevance of our research, we conducted our studies in the management domain.

### Fear Motives, Intrusive Thoughts, and Self-Control Strategies

Several researchers suggest differentiating motivational constructs such as goals into approach (i.e., hope) and avoidance (i.e., fear) tendencies [20,21,22]. In this regard, motives are not an exception [23,24]. In the motive literature, approach tendencies are termed as hope motives and avoidance tendencies are termed fear motives. 

Research shows that there are relatively stable individual differences in anxiety-proneness. To describe these differences, various constructs have been introduced, for instance, avoidance tendency [20], trait anxiety [25], and dispositional fear motives [25,26]. Despite minor differences [26], we use these constructs interchangeably in this research to mean “relatively stable individual differences in anxiety-proneness” [27]. In doing so, we do not focus on what the causes of these constructs are. However, we focus on how these constructs exert influence on other constructs such as intrusive thoughts. Based on attentional control theory [16], we speculate that these constructs interfere with the inhibitory function of the inhibitory system and thus, use these constructs interchangeably. There is empirical evidence that fear motives and trait anxiety are predisposing factors for negative affect [13,28,29], a higher bias for negative information [30], impaired intrinsic motivation [31,32] distress and distress tolerance [33,34] as well as depression [24,35]. 

Researchers have shown that anxiety is positively associated with intrusive thoughts [15,16,18] which are defined as repeated, spontaneous, and involuntary memories or images that come to individuals’ minds [36]. According to attentional control theory [16], anxiety interferes with the inhibition function that helps individuals stay on track with their goals by inhibiting impulses to stimuli, which results in increased intrusive thoughts. Because anxiety and fear motives might have similar effects on the inhibitory function, this led us to predict that fear motives may also interfere with the inhibition function and thus are positively associated with intrusive thoughts. 

Individuals experiencing intrusive thoughts use attentional resources to suppress these thoughts, which reduces a person’s attentional resources [18,37] and may bring about difficulties in subsequent self-control activities [38,39,40]. According to Baumeister and colleagues’ strength model of self-control, self-control draws on a limited reservoir, and with individuals’ engagement in self-control activities, they are likely to fail at self-control in the subsequent self-control activities as the reservoir becomes depleted. Recently, the strength model of self-control has been criticized by a number of researchers. Results from several large multilab replication studies did not find evidence in support of the strength model of self-control, specifically ego depletion [41,42]. However, some others found supporting evidence for the model [43,44]. What is more, some researchers discuss that although the controlled laboratory studies testing this idea may have failed to yield reliable and robust effects, the general theoretical idea might have some merits [45]. In support of this, field studies have documented promising results showing failures in overcoming an easy task after initial high demands [46,47]. Another influential model that explains self-control failure is the integrative self-control theory [48]. The model posits that self-control failure depends on individuals’ motivation or willingness to exert self-control to inhibit impulses. For our purposes, whether intrusive thoughts consume resources that make it difficult for individuals to exercise self-control in subsequent tasks or they demotivate individuals to exercise self-control to inhibit impulses is not of central importance. Regardless, it seems that suppression of intrusive thoughts requires self-control, which may be lacking for other activities requiring self-control. Due to this, we claim that there is a negative relationship between intrusive thoughts and the frequency with which individuals exercise self-control strategies. 

From a functional perspective, self-control strategies may be defined as a set of strategies individuals employ to support action tendencies against competing behavioral impulses [28,49,50]. Self-control strategies might include motivation control (i.e., developing positive goal-related fantasies in the face of difficulties; Refs. [51,52]), emotion control (i.e., adjusting one’s emotional states to the demands of the current intention; cf. [53,54]), attention control (i.e., focusing attention on aspects of the situation relevant for implementing the current intention; cf. [55,56]), and decision control (i.e., employing mechanisms to decide quickly and avoid rumination; cf. [57,58]). Researchers have found that employing self-control strategies might be positively related to positive affect [59] and subjective well-being [60] as self-control strategies reduce goal conflicts and suppress competing behavioral impulses. Following this, we also propose a positive relationship between self-regulation strategies and positive affect. 

Summing up the evidence, the general proposition of this research is that fear motives are expected to instigate unwanted intrusive thoughts that require self-control to suppress. In turn, the exercise of self-control impedes the use of self-control strategies that might be necessary for other tasks. In addition, we propose that employing self-control strategies is positively associated with positive affect. More importantly, these lead us, further, to propose that intrusive thoughts mediate the relationship between fear motives and self-control strategies, and intrusive thoughts and self-control strategies serially mediate the relationship between fear motives and positive affect. Two studies were conducted to test these propositions. To secure the external validity of the research, data were collected from managers of diverse companies.

## 2. Materials and Methods

### 2.1. Study 1

A survey link was sent to 112 managers from various German industry and trade companies, 103 of whom answered the questionnaire. Due to missing data, three participants were excluded, resulting in a final sample of 100 managers at middle and lower management levels. Participants were given confidential feedback on their personal results in exchange for filling out the questionnaires. All participants were Caucasian (24 females) with ages ranging from 28 to 60 (*M* = 41.8, *SD* = 7.7).

### 2.2. Study 2

Using the same recruitment procedure-but different companies-as in Study 1, 113 managers were invited to the online survey, 83 of whom filled out questionnaires for the respective measures at both Time 1 and Time 2. Due to missing data, three participants were excluded. The demographic background of the remaining 80 (21 females) participants was similar to that of participants in Study 1, with ages ranging from 22 to 61 (*M* = 38.8, *SD* = 8.2). 

### 2.3. Procedure 

Data were collected in one measurement point for Study 1 and two measurement points with a time span of approximately five months between the first (Time 1) and second (Time 2) data collection for Study 2. For Study 2, data on fear motives and positive affect were collected at Time 1, and intrusive thoughts, self-control strategies, and positive affect were collected at Time 2. 

### 2.4. Measures

#### 2.4.1. Fear Motives (Study 1 and Study 2)

Implicit fear motives were measured with the Multi-Motive-Grid (MMG; Ref. [24]), a semi-projective instrument less transparent to respondents than direct self-reports of fear. The MMG employs pictorial stimulus material to arouse participants’ implicit motives. It includes 14 pictures, each accompanied by a set of statements. Each statement represents one of the three motive domains: achievement, affiliation, and power. Participants are asked whether each statement fits a corresponding picture on a bipolar “yes/no” scale. Each motive domain is composed of approach (hope for achievement, affiliation, and power) and avoidance (fear of achievement, affiliation, and power) facets. Example statements for each fear motive domain includes “Being afraid of being overpowered by other people” (fear of power), “Being afraid of being rejected by others” (fear of rejection), and “Thinking about lack of abilities at this task” (fear of failure). Sokolowski et al. [24] reviewed several studies demonstrating high internal consistency, reliability, predictive validity, and discriminative validity of the MMG. The three fear motive domain scores were aggregated to obtain a comprehensive fear motives score (36 items). Cronbach’s alphas were 0.81 and 0.83 for Study 1 and Study 2, respectively.

#### 2.4.2. Intrusive Thoughts (Study 1 and Study 2)

Intrusive thoughts were measured with a four-item intrusions scale of the German version of the Volitional Components Inventory (VCI; Refs. [61,62]). The participants were asked to indicate how often they experienced the situation/process portrayed in each item recently on a 7-point Likert scale, ranging from 1 (“very rarely”) to 7 (“very often”). A sample item is “Having insuppressible disturbing thoughts”. Cronbach’s alphas for this scale were 0.89 and 0.91 for Study 1 and Study 2, respectively. 

#### 2.4.3. Self-Control Strategies (Study 1 and Study 2)

Four subscales from the German version of the Volitional Components Inventory (VCI; Refs. [61,62]) were used to measure self-control strategies: motivation control, emotion control, attention control, and decision control. Each subscale consists of six items. In both studies, we used five items to measure attention control. The participants were asked to indicate how often they experienced a situation/process portrayed in each item recently on a 7-point Likert scale ranging from 1 (“very rarely”) to 7 (“very often”). Sample items include “Trying consciously to keep my attention stable” (attention control), “Cheering myself up to make things work” (emotion control), “Considering positive incentives concerning the matter” (motivation control), and “Having no difficulties with spontaneous decisions” (decision control). The subscales were aggregated into a composite measure of self-control strategies. Cronbach’s alphas were 0.92 and 0.91 for Study 1 and Study 2, respectively. 

#### 2.4.4. Positive Affect (Study 2)

Positive affect was measured only in Study 2 using the 8-item instrument introduced by Brunstein et al. [63]. Participants read, “How often have you recently experienced the following moods…” and then rated emotional adjectives on a seven-point scale from 1 (“never”) to 7 (“very frequently”). Positive affect was assessed using an aggregated measure of the elated mood (happy, joyful, pleased, and excellent) and the activation subscale (energetic, active, cheery, and vigorous). Cronbach’s alphas were 0.91 and 0.93 for Time 1 and Time 2, respectively. 

### 2.5. Analyses

To test the predictions, we employed Bayesian mediation analysis [64] for parameter estimation. We used Bayesian mediation analysis as an analytic tool because it has some advantages over conventional frequentist mediation analyses. Firstly, parameter interpretation is more natural and intuitive. Unlike the frequentist approach, Bayesian analysis provides information about credible parameter values (e.g., indirect effects) given the observed data [65]. Second, Bayesian mediation analyses construct credible intervals for indirect effects without imposing any restrictive normality assumptions, which is especially important for small samples [64]. Moreover, it is possible to incorporate findings from meta-analyses, previous studies, or pilot studies into Bayesian mediation analyses, which might result in a higher power for small samples [66]. We used the PyMC Python package [67] with the recommended four MCMC sampling chains and 4000 iterations for each chain. All of the analyses achieved sufficient model convergence (all R-hat values = 1.00). We provide trace plots for model convergence in Supplemental Materials (see Figures S1–S3). 

#### 2.5.1. Model Specification

For Study 1, we conducted a Bayesian mediation model with fear motives as the predictor, intrusive thoughts as the mediator, and self-control strategies as the outcome variable. For Study 2, we, first of all, replicated the findings of Study 1 using the same strategy (i.e., fear motives measured at T1 as the predictor, intrusive thoughts measured at T2 as the mediator, and self-control strategies measured at T2 as the outcome variable). Finally, we extended Study 1 by adding positive affect measured at T2 as the outcome variable and positive affect measured at T1 as a control variable in the mediation model specified. 

#### 2.5.2. Prior Selection

Following recommendations by Gelman et al. [68], we used weakly informative priors for regression coefficients and intercepts (a normal distribution with µ = 0 and σ = 1) in Study 1. In Study 2, we used the posterior’s means and variances from Study 1 as priors for regression coefficients and intercepts. However, as the mediator and outcome variable in Study 2 were measured at T2, we inflated the variance in the priors by three so that these priors do not dominate the data [64]. Finally, for the priors of the variables not included in Study 1, we used the weakly informative priors as in Study 1. The details of the modeling can be found in the Python code provided in Supplemental Materials as well as on Open Science Framework (see https://osf.io/mvcet/; accessed on 24 January 2023). 

## 3. Results

### 3.1. Descriptive Statistics and Correlations

Table 1 displays descriptive statistics of and correlations among study variables. As expected, fear motives were positively associated with intrusive thoughts, and negatively associated with self-control strategies and positive affect. Additionally, intrusive thoughts, self-control strategies, and positive affect were substantially correlated. 

### 3.2. Hypothesis Testing

In the following, we provide parameters with the 95% highest density interval (HDI), which indicates 95% of the most probable parameter values given the observed data [69]. The parameter can be considered credible if 0 is not included in the 95% HDI [65].

**Figure 1:**
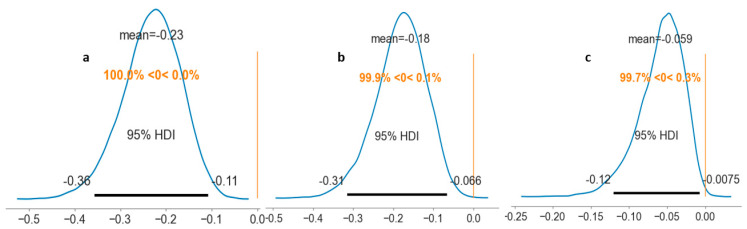
Distributions show the probability density for the indirect effects. Panel (**a**,**b**) show the probability density for the indirect effect of fear motives on self-control strategies through intrusive thoughts in Study 1 and Study 2, respectively. Panel (**c**) shows the probability density for the indirect effect of fear motives on positive affect serially through intrusive thoughts and self-control strategies in Study 2. HDI = highest density interval.

The results from Studies 1 and 2 revealed credible evidence for a positive effect of fear motives on intrusive thoughts, b = 0.40, SE = 0.09, 95% HDI = [0.22, 0.58] and b = 0.33, SE = 0.10, 95% HDI = [0.14, 0.53] respectively. That is, there was a significant positive relationship between fear motives and intrusive thoughts in both Study 1 and Study 2, which supports predictions. Furthermore, in both studies, there was credible evidence for a negative effect of intrusive thoughts on self-control strategies, b = −0.58, SE = 0.09, 95% HDI = [−0.75, −0.40] and b = −0.55, SE = 0.09, 95% HDI = [−0.74, −0.37] respectively, supporting our predictions that intrusive thoughts and self-control strategies are negatively associated. Moreover, in support of the predictions, the indirect effect of fear motives on self-control strategies through intrusive thoughts was found to be credible, Est. = −0.23, SE = 0.06, 95% HDI = [−0.36, −0.11] and Est. = −0.18, SE = 0.06, 95% HDI = [−0.31, −0.07], respectively. That is, in both studies, we found there is an indirect effect of fear motives on self-regulation strategies through intrusive thoughts. For further details, see Table 2 and Figure 1. 

In addition, Study 2 results demonstrated credible evidence for a positive effect of self-control strategies on positive affect, b = 0.32, SE = 0.11, 95% HDI = [0.10, 0.55]. Similarly, in line with predictions, there was credible evidence for an indirect effect of fear motives on positive affect serially through intrusive thoughts and self-control strategies, b = −0.06, SE = 0.03, 95% HDI = [−0.12, −0.01]. For further details, see Table 2 and Figure 1.

## 4. Discussion

This study started with the speculation that individuals high on trait anxiety or fear motives display affective, cognitive, and behavioral responses hinting at poor skills in self-control [26]. Although this speculation seemed plausible, to our knowledge, there is no empirical investigation putting this speculation to a test. With this research, we wanted to close this research gap and investigate if fear motives are positively associated with intrusive thoughts and if intrusive thoughts, in turn, are negatively associated with self-control strategies. Additionally, we tested the previously documented positive relationship between self-control strategies and positive affect [70]. More importantly, we tested if the relationships between fear motives and self-control strategies, and fear motives and positive affect are mediated by intrusive thoughts, and serially by intrusive thoughts and self-control strategies respectively. We employed Bayesian mediation analysis and the results supported our predictions.

Specifically, we expected a positive relationship between fear motives and intrusive thoughts. Deriving from attentional control theory [15,16], we expected fear motives to have a similar function as trait anxiety on inhibition function. That is, we expected fear motives to interfere with the inhibiting function of the inhibitory system and, thus, lead to unwanted intrusive thoughts. In line with the literature on trait anxiety and our expectation [18], we found a positive relationship between fear motives and intrusive thoughts. Additionally, we expected a negative relationship between intrusive thoughts and self-control strategies as individuals who have highly intrusive thoughts might use cognitive and attentional resources to suppress them [18]. This use of resources might deplete such resources, which brings about difficulties in exerting further self-control such as employing self-control strategies. Our findings were in line with this expectation. Further, we expected a negative relationship between fear motives and self-control strategies and this relationship to be mediated by intrusive thoughts with theoretical and empirical reasoning presented. We found supporting evidence that fear motives are negatively associated with self-control strategies. Given that one important self-control strategy studied is cognitive control and the others require cognitive processing, these findings are in line with the previous literature showing that neuroticism is negatively associated with cognition [37]. In a similar vein, we found evidence that intrusive thoughts are the mediating mechanisms for the relationship between fear motives and self-control strategies. As postulated by attentional control theory [15,16], the findings demonstrated that fear motives are indirectly associated with self-control strategies through intrusive thoughts. These findings were obtained in both Studies 1 and 2. 

In Study 2, we also wanted to extend the findings from Study 1 by investigating if self-control strategies are positively associated with positive affect and if intrusive thoughts and self-control strategies serially mediate the relationship between fear motives and positive affect. The results showed a positive relationship between self-control strategies and positive affect. Previous studies in this line demonstrated that trait self-control and self-control strategies have positive relationships with subjective well-being and positive affect e.g., [60]. Our results replicate these findings. Finally, previous empirical studies showed that fear motives are positively associated with negative moods [11]. Depending on the theoretical stipulations and previous empirical findings, we expected and found the negative relationship between fear motives and positive affect to be serially mediated by intrusive thoughts and self-control strategies.

### 4.1. Theoretical Implications

The findings confirm the central proposition of this research that fear motives are negatively associated with the frequency with which individuals employ self-control strategies. This is consistent with Kanfer and Heggestad’s [26] notion that people high on fear motives have impaired self-regulation. Fear motives, conceptualized as relatively stable and enduring tendencies [26] require self-control to suppress intrusive thoughts, which is associated with difficulties to employ further self-control strategies. The findings here were obtained in field research with managers and sustain the ecological validity of the notion of ego depletion [38,40]. 

Moreover, both studies showed that fear motives are significantly associated with intrusive thoughts. This is in line with the previous literature showing that trait anxiety might be a cause of intrusive thoughts as it interferes with the inhibition of inhibition function [15,18]. This pattern of results also shows that fear motives and trait anxiety might be similar relationships with executive function or inhibition function. 

Finally, the validity of our findings is strengthened owing to two methodological aspects of the studies. Firstly, most of the previous studies on anxiety relied on self-report measures [25]. By utilizing a semi-projective measure of fear motives, we suggest the findings to be more robust as the measure is less transparent to respondents than typical self-report measures. Second, recent discussions around self-control failure suggest that the experimental findings might not do the self-control failure concept a favor and fall short of producing reliable and robust effects [42]. By collecting data from an applied field, we follow the previous literature [48] and support the notion that tasks that require high demands lead to impairments in subsequent behaviors. 

### 4.2. Practical Implications

This research is important because of the scant data on the relationship between fear motives and self-control strategies in applied and clinical settings. It shows that people in management may suffer from dispositional fear by experiencing increments in intrusive thoughts and subsequent impediments to self-control and well-being. It would be interesting to see if these findings can be replicated using non-managerial samples in the field. Furthermore, our findings suggest that fear motives might bring about negative consequences such as heightened intrusive thoughts and less frequent employment of self-regulation strategies. This might make it essential to attend to the fear motives and find ways to cope with them to prevent them from leading to these negative experiences. 

### 4.3. Limitations and Directions for Future Research

This study is not free of its limitations. A major limitation of our study is its correlational design. As we described the processes by which fear motives are associated with self-regulation strategies and positive affect, it is essential to employ experimental design to manipulate these processes and observe their effects on the outcome variables. This will establish the causal link implied but not tested between the study variables. Thus, future studies should experimentally test the links between our variables. The second limitation of the study is that we did not measure some variables that are assumed in our model. For example, the theoretical suggestions that fear motives interfere with inhibitory function and, thus, lead to intrusive thoughts suggest that fear motives are negatively related to inhibition of inhibitory function. However, without measuring and testing this assumption, we may not be able to make strong claims. Future studies should measure the assumed variables and replicate our findings with these variables included in their model. Finally, another limitation of our research is the small sample size. Although we utilized Bayesian statistics to account for the limitations of typical mediation analysis with small sample sizes, future studies would benefit from larger sample sizes in terms of inferential aims. 

## Figures and Tables

**Table 1:** Means, standard deviations, and correlations among study variables.

Variable	*M*	*SD*	1	2	3	4	5	6
***Study 1***								
1. Sex (women = 0)	-	-						
2. Age	41.84	7.74	0.04					
3. Fear motives	12.80	5.75	−0.14	−0.04				
4. Intrusive thoughts	3.36	1.28	−0.21 *	−0.13	0.40 **			
5. Self-regulation strategies	4.48	0.79	0.18	0.10	−0.30 **	−0.61 **		
***Study 2***								
1. Sex (women = 0)	-	-						
2. Age	38.80	8.24	0.11					
3. Fear motives	10.71	6.63	−0.24 *	−0.21				
4. Intrusive thoughts	3.06	1.18	0.02	−0.08	0.32 **			
5. Self-regulation strategies	4.98	0.70	0.04	0.16	−0.23 *	−0.56 **		
6. Positive affect T1	5.06	0.95	−0.02	−0.04	−0.07	−0.42 **	0.52 **	
7. Positive affect T2	5.30	1.00	−0.17	0.14	−0.25 *	−0.60 **	0.55 **	0.31 **

Note. Study 1 *N* = 100, Study 2 *N* = 80. *M* and *SD* are used to represent mean and standard deviation, respectively. * indicates *p* < 0.05. ** indicates *p* < 0.01.

**Table 2:** Bayesian Mediation Models for Studies 1 and 2.

	Intrusive Thoughts		Self-Control Strategies		Positive Affect	
Variable	Estimate (SE)	95% HDI	Estimate (SE)	95% HDI	Estimate (SE)	95% HDI
***Study 1***						
Intercept	0.0(0.09)	[−0.18, 0.18]	−0.0(0.08)	[−0.16, 0.16]		
Fear Motives	0.40(0.09)	[0.22, 0.58]	−0.06(0.09)	[−0.23, 0.11]		
Intrusive thoughts	-	-	−0.58(0.09)	[−0.75, −0.40]		
						
*Indirect effect*			−0.23(0.06)	[−0.36, −0.11]		
*Total effect*			−0.30(0.10)	[−0.48, −0.10]		
***Study 2***						
Intercept	0.0(0.01)	[−0.20, 0.20]	0.0(0.09)	[−0.17, 0.17]	0.00(0.09)	[−0.17, 0.17]
Fear motives	0.33(0.10)	[0.14, 0.53]	−0.06(0.09)	[−0.23, 0.14]	−0.04(0.09)	[−0.23, 0.14]
Intrusive thoughts	-	-	−0.55(0.09)	[−0.74, −0.37]	−0.42(0.11)	[−0.64, −0.21]
Self-control S.			-	-	0.32(0.11)	[0.10, 0.55]
Positive affect T1			-	-	−0.03(0.10)	[−0.23, 0.17]
						
*Indirect effect*			−0.18(0.06)	[−0.31, −0.07]	−0.06(0.03)	[−0.12, −0.01]
*Total effect*			−0.24(0.11)	[−0.44, −0.03]	−0.10(0.10)	[−0.29, 0.09]

Note. HDI = highest density interval.

## Data Availability

The datasets and the codes for data analyses for the current study are made publicly available on Open Science Framework (see https://osf.io/mvcet/; accessed on 24 January 2023).

## Supplementary Materials

The following supporting information can be downloaded at: https://www.mdpi.com/article/10.3390/bs13050384/s1.

## References

[1] Lang J.W.B., Zettler I., Ewen C., Hülsheger U.R. (2012). Implicit motives, explicit traits, and task and contextual performance at work. J. Appl. Psychol..

[2] Runge J.M., Lang J.W.B., Zettler I., Lievens F. (2020). Predicting counterproductive work behavior: Do implicit motives have incremental validity beyond explicit traits?. J. Res. Personal..

[3] Schultheiss O.C., Jones N.M., Davis A.Q., Kley C. (2008). The role of implicit motivation in hot and cold goal pursuit: Effects on goal progress, goal rumination, and emotional well-being. J. Res. Personal..

[4] McClelland D.C. (1987). Human Motivation.

[5] Bakaç C., Kehr H.M. (2023). Getting to the bargaining table: The role of explicit motives and traits in negotiation initiation. Personal. Individ. Differ..

[6] Kehr H.M. (2004). Integrating implicit motives, explicit motives, and perceived abilities: The compensatory model of work motivation and volition. Acad. Manag. Rev..

[7] Thrash T.M., Elliot A.J. (2002). Implicit and self-attributed achievement motives: Concordance and predictive validity. J. Personal..

[8] Schönbrodt F.D., Gerstenberg F.X.R. (2012). An IRT analysis of motive questionnaires: The Unified Motive Scales. J. Res. Personal..

[9] Winter D.G. (2010). Why achievement motivation predicts success in business but failure in politics: The importance of personal control. J. Personal..

[10] Wolff H.G., Weikamp J.G., Batinic B. (2018). Implicit motives as determinants of networking behaviors. Front. Psychol..

[11] Langens T.A., Schüler J. (2005). Written emotional expression and emotional well-being: The moderating role of fear of rejection. Personal. Soc. Psychol. Bull..

[12] Wegner M., Wieland A., Mempel G. (2017). The implicit fear of power motive is associated with practice time in elite karateka and tennis players. Int. J. Sport Exerc. Psychol..

[13] Langens T.A., Schmalt H.D. (2002). Emotional consequences of positive daydreaming: The moderating role of fear of failure. Personal. Soc. Psychol. Bull..

[14] Gable S.L., Reis H.T., Elliot A.J. (2003). Evidence for bivariate systems: An empirical test of appetition and aversion across domains. J. Res. Personal..

[15] Eysenck M.W., Derakshan N. (2011). New perspectives in attentional control theory. Personal. Individ. Differ..

[16] Eysenck M.W., Derakshan N., Santos R., Calvo M.G. (2007). Anxiety and cognitive performance: Attentional control theory. Emotion.

[17] Beadel J.R., Green J.S., Hosseinbor S., Teachman B.A. (2013). Influence of age, thought content, and anxiety on suppression of intrusive thoughts. J. Anxiety Disord..

[18] Stawski R.S., Sliwinski M.J., Smyth J.M. (2006). Stress-related cognitive interference predicts cognitive function in old age. Psychol. Aging.

[19] Heckhausen J. (2011). The motivation-volition divide and its resolution in action-phase models of developmental regulation. Res. Hum. Dev..

[20] Atkinson J.W. (1964). An Introduction to Motivation.

[21] Carver C.S., Lawrence J.W., Scheier M.F. (1999). Self-discrepancies and affect: Incorporating the role of feared selves. Personal. Soc. Psychol. Bull..

[22] McClelland D.C., Atkinson J.W., Clark R.A., Lowell E.L. (1953). The Achievement Motive.

[23] Schmalt H., Heckhausen H., Heckhausen J., Heckhausen H. (2008). Power motivation. Motivation and Action.

[24] Sokolowski K., Schmalt H.D., Langens T.A., Puca R.M. (2000). Assessing achievement, affiliation, and power motives all at once: The multi-motive grid (MMG). J. Personal. Assess..

[25] Twenge J.M. (2000). The age of anxiety? Birth cohort change in anxiety and neuroticism, 1952-1993. J. Personal. Soc. Psychol..

[26] Kanfer R., Heggestad E.D., Cummings L.L., Staw B.M. (1997). Motivational Traits and Skills: A Person-Centered Approach to Work Motivation. Research in Organizational Behavior.

[27] Spielberger C.D., Rickman R.L., Sartorius N., Andreoli V., Cassano G., Eisenberg L., Kielkolt P., Pancheri P., Racagni G. (1990). Assessment of State and Trait Anxiety. Anxiety: Psychobiological and Clinical Perspectives.

[28] Sokolowski K. (1993). Emotion und Volition [Emotion and Volition].

[29] Stroe S., Shepherd D., Wincent J. (2018). Fear of failure and negative affect in early stage entrepreneurs: Regulation through passion. Acad. Manag. Proc..

[30] Lerche V., Bucher A., Voss A. (2019). Processing emotional expressions under fear of rejection: Findings from diffusion model analyses. Emotion.

[31] Elliot A.J., Harackiewicz J.M. (1996). Approach and avoidance achievement goals and intrinsic motivation: A mediational analysis. J. Personal. Soc. Psychol..

[32] Sun J.C.Y., Syu Y.R., Lin Y.Y. (2017). Effects of conformity and learning anxiety on intrinsic and extrinsic motivation: The case of Facebook course groups. Univers. Access Inf. Soc..

[33] Allan N.P., Macatee R.J., Norr A.M., Raines A.M., Schmidt N.B. (2015). Relations between common and specific factors of anxiety sensitivity and distress tolerance and fear, distress, and alcohol and substance use disorders. J. Anxiety Disord..

[34] Emmons R.A., Gollwitzer P.M., Bargh J.A. (1996). Striving and Feeling: Personal Goals and Subjective Well-Being. The Psychology of Action: Linking Cognition and Motivation to Behavior.

[35] Wang T., Li M., Xu S., Liu B., Wu T., Lu F., Xie J., Peng L., Wang J. (2019). Relations between trait anxiety and depression: A mediated moderation model. J. Affect. Disord..

[36] Trinder H., Salkovskis P.M. (1994). Personally relevant intrusions outside the laboratory: Long-term suppression increases intrusion. Behav. Res. Ther..

[37] Munoz E., Sliwinski M.J., Smyth J.M., Almeida D.M., King H.A. (2013). Intrusive thoughts mediate the association between neuroticism and cognitive function. Personal. Individ. Differ..

[38] Baumeister R.F., Bratslavsky E., Muraven M., Tice D.M. (1998). Ego depletion: Is the active self a limited resource?. J. Personal. Soc. Psychol..

[39] Baumeister R.F., Vohs K.D., Tice D.M. (2007). The strength model of self-control. Curr. Dir. Psychol. Sci..

[40] Muraven M., Baumeister R.F. (2000). Self-regulation and depletion of limited resources: Does self-control resemble a muscle?. Psychol. Bull..

[41] Hagger M.S., Chatzisarantis N.L.D., Alberts H., Anggono C.O., Batailler C., Birt A.R., Brand R., Brandt M.J., Brewer G.A., Bruyneel S. (2016). A multilab preregistered replication of the ego-depletion effect. Perspect. Psychol. Sci..

[42] Vohs K.D., Schmeichel B.J., Lohmann S., Gronau Q.F., Finley A.J., Ainsworth S.E., Alquist J.L., Baker M.D., Brizi A., Bunyi A. (2021). A multisite preregistered paradigmatic test of the ego-depletion effect. Psychol. Sci..

[43] Dang J., Barker P., Baumert A., Bentvelzen M., Berkman E., Buchholz N., Buczny J., Chen Z., De Cristofaro V., de Vries L. (2021). A multilab replication of the ego depletion effect. Soc. Psychol. Personal. Sci..

[44] Lin H., Saunders B., Friese M., Evans N.J., Inzlicht M. (2020). Strong effort manipulations reduce response caution: A preregistered reinvention of the ego-depletion paradigm. Psychol. Sci..

[45] Friese M., Loschelder D.D., Gieseler K., Frankenbach J., Inzlicht M. (2019). Is ego depletion real? An analysis of arguments. Personal. Soc. Psychol. Rev..

[46] Linder J.A., Doctor J.N., Friedberg M.W., Reyes Nieva H., Birks C., Meeker D., Fox C.R. (2014). Time of day and the decision to prescribe antibiotics. JAMA Intern. Med..

[47] Sonnentag S., Jelden S. (2009). Job stressors and the pursuit of sport activities: A day-level perspective. J. Occup. Health Psychol..

[48] Kotabe H.P., Hofmann W. (2015). On integrating the components of self-control. Perspect. Psychol. Sci..

[49] Kuhl J., Boekarts M., Pintrich P.R., Zeidner M. (2000). A Functional-Design Approach to Motivation and Self-Regulation: The Dynamics of Personality Systems and Interactions. Handbook of Self-Regulation.

[50] Kuhl J., Goschke T., Kuhl J., Beckmann. J. (1994). A Theory of Action Control: Mental Subsystems, Modes of Control, and Volitional Conflict-Resolution Strategies. Volition and Personality: Action Versus State Orientation.

[51] Mischel W., Bargh J.A., Gollwitzer P.M. (1996). From Good Intentions to Willpower. The Psychology of Action: Linking Cognition and Motivation to Behavior.

[52] Oettingen G., Pak H., Schnetter K. (2001). Self-regulation of goal-setting: Turning free fantasies about the future into binding goals. J. Personal. Soc. Psychol..

[53] Bagozzi R.P., Baumgartner H., Pieters R. (1998). Goal-directed emotions. Cogn. Emot..

[54] Gross J.J. (1999). Emotion regulation: Past, present, future. Cogn. Emot..

[55] Atkinson J.W., Birch D.A. (1970). A Dynamic Theory of Action.

[56] Egeth H.E., Yantis S. (1997). Visual attention: Control, representation, and time course. Annu. Rev. Psychol..

[57] Koole S.L., Smeets K., van Knippenberg A., Dijksterhuis A. (1999). The cessation of rumination through self-affirmation. J. Personal. Soc. Psychol..

[58] Tyszka T. (1998). Two pairs of conflicting motives in decision making. Organ. Behav. Hum. Decis. Process..

[59] Wenzel M., Rowland Z., Kubiak T. (2021). Examining five pathways on how self-control is associated with emotion regulation and affective well-being in daily life. J. Personal..

[60] Nielsen K.S., Gwozdz W., De Ridder D. (2019). Unraveling the relationship between trait self-control and subjective well-being: The mediating role of four self-control strategies. Fron. Psychol..

[61] Kuhl J., Fuhrmann A. (1997). Das Selbststeuerungs-Inventar (SSI).

[62] Kuhl J., Fuhrmann A., Heckhausen J., Dweck C.S. (1998). Decomposing Self-Regulation and Self-Control: The Volitional Components Inventory. Motivation and Self-Regulation Across the Life Span.

[63] Brunstein J.C., Lautenschlager U., Nawroth B., Pöhlmann K. (1995). Persönliche Anliegen, soziale Motive und emotionales Wohlbefinden [Personal goals, social motives, and emotional well-being]. Z. Differ. Diagn. Psychol..

[64] Yuan Y., MacKinnon D.P. (2009). Bayesian mediation analysis. Psychol. Met..

[65] Kruschke J.K., Aguinis H., Joo H. (2012). The time has come: Bayesian methods for data analysis in the organizational cciences. Organ. Res. Methods.

[66] Miočević M., MacKinnon D.P., Levy R. (2017). Power in Bayesian mediation analysis for small sample research. Struct. Equ. Model..

[67] Patil A., Huard D., Fonnesbeck C.J. (2010). PyMC: Bayesian Stochastic modelling in Python. J. Stat. Softw..

[68] Gelman A., Carlin J.B., Stern H.S., Dunson D.B., Vehtari A., Rubin D.B. (2013). Bayesian Data Analysis.

[69] Kruschke J.K., Liddell T.M. (2018). The Bayesian new statistics: Hypothesis testing, estimation, meta-analysis, and power analysis from a Bayesian perspective. Psychon. Bull. Rev..

[70] Ronen T., Hamama L., Rosenbaum M., Mishely-Yarlap A. (2016). Subjective well-being in adolescence: The role of self-control, social support, age, gender, and familial crisis. J. Happiness Stud..

