# Supplementary material for: Unpacking the Relationship between Fear Motives and Self-Control Strategies among Managers: The Mediating Role of Intrusive Thoughts
Source: Behav Sci (Basel). 2023 May 6;13(5):384. doi: 10.3390/bs13050384 (PMC10215596; doi:10.3390/bs13050384)
Supplement: Supplementary file 1 [file behavsci-13-00384-s001.zip › behavsci-2296362-supplementary.pdf]

Supplemental Materials

Unpacking the Relationship between Fear Motives and Self-Control Strategies among Managers: The Mediating Role of Intrusive Thoughts

Table of Contents

Trace Plot for convergence – (DV: Self-Control Strategies in Study 1) 2

Trace Plot for convergence – (DV: Self-Control Strategies and Positive Affect in Study 2) 4

Python Code for Analyses 7

Trace Plot for convergence – (DV: Self-Control Strategies in Study 1)

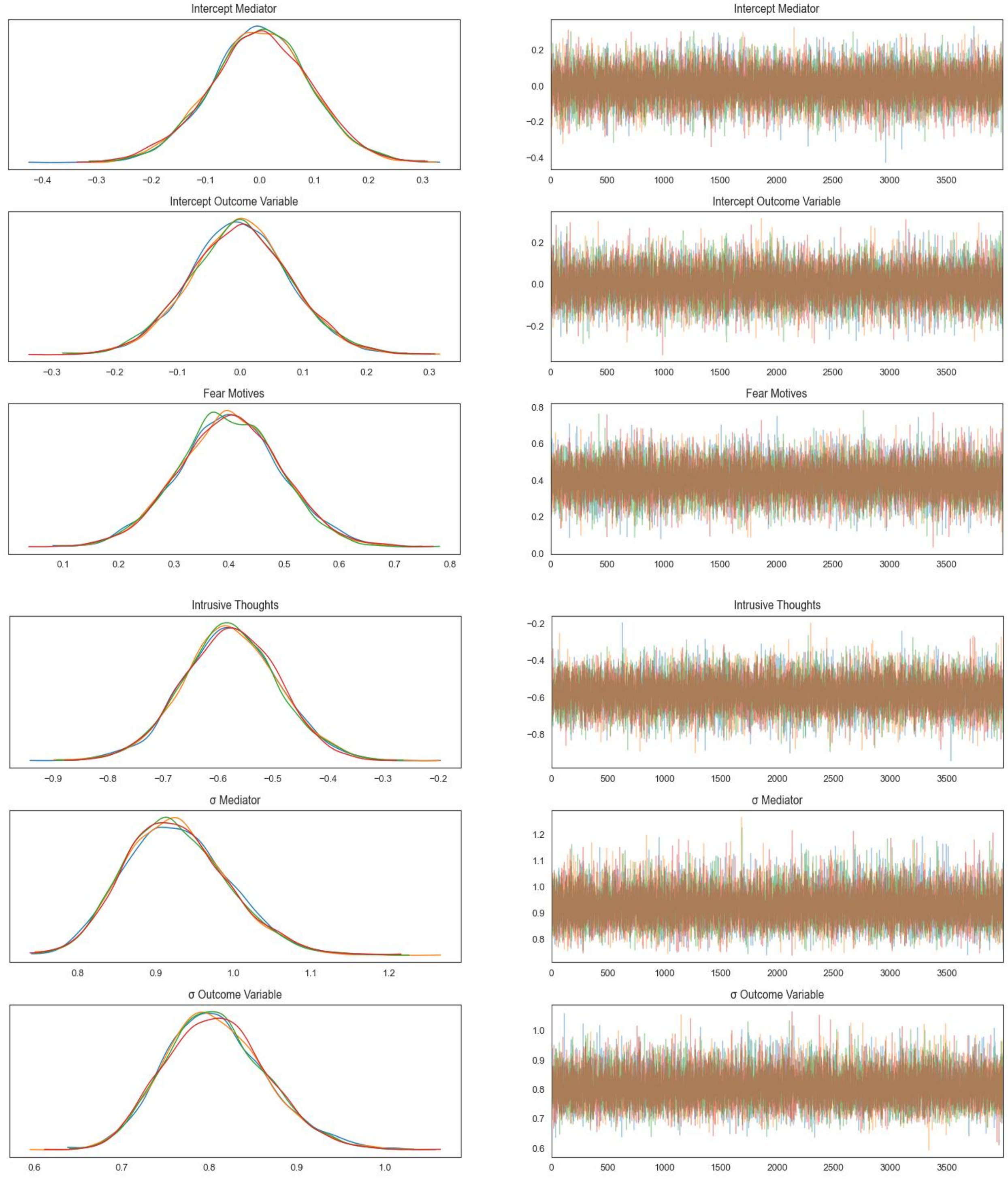

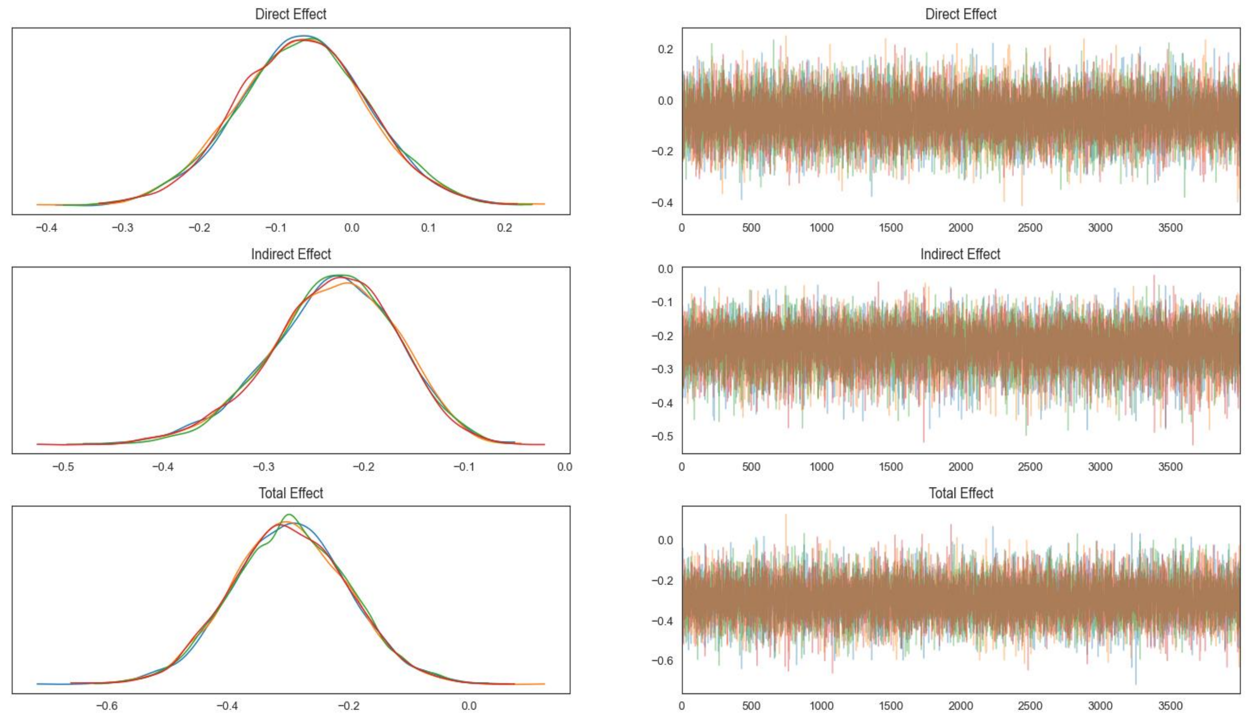

Figure S1. Trace plots for the Bayesian mediation model for testing the indirect effect of fear motives on self-control strategies through intrusive thoughts in Study 1.

Trace Plot for convergence – (DV: Self-Control Strategies and Positive Affect in Study 2)

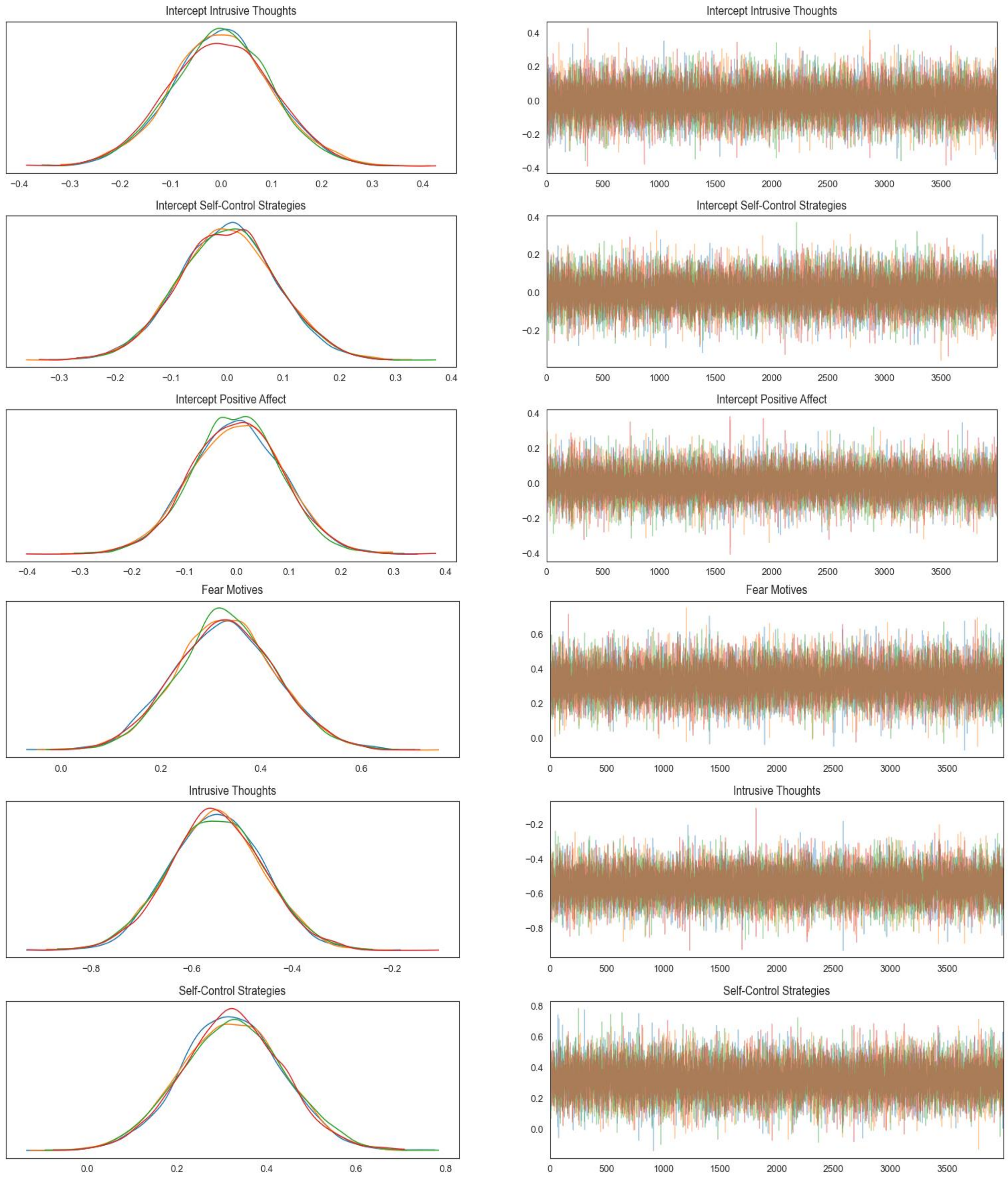

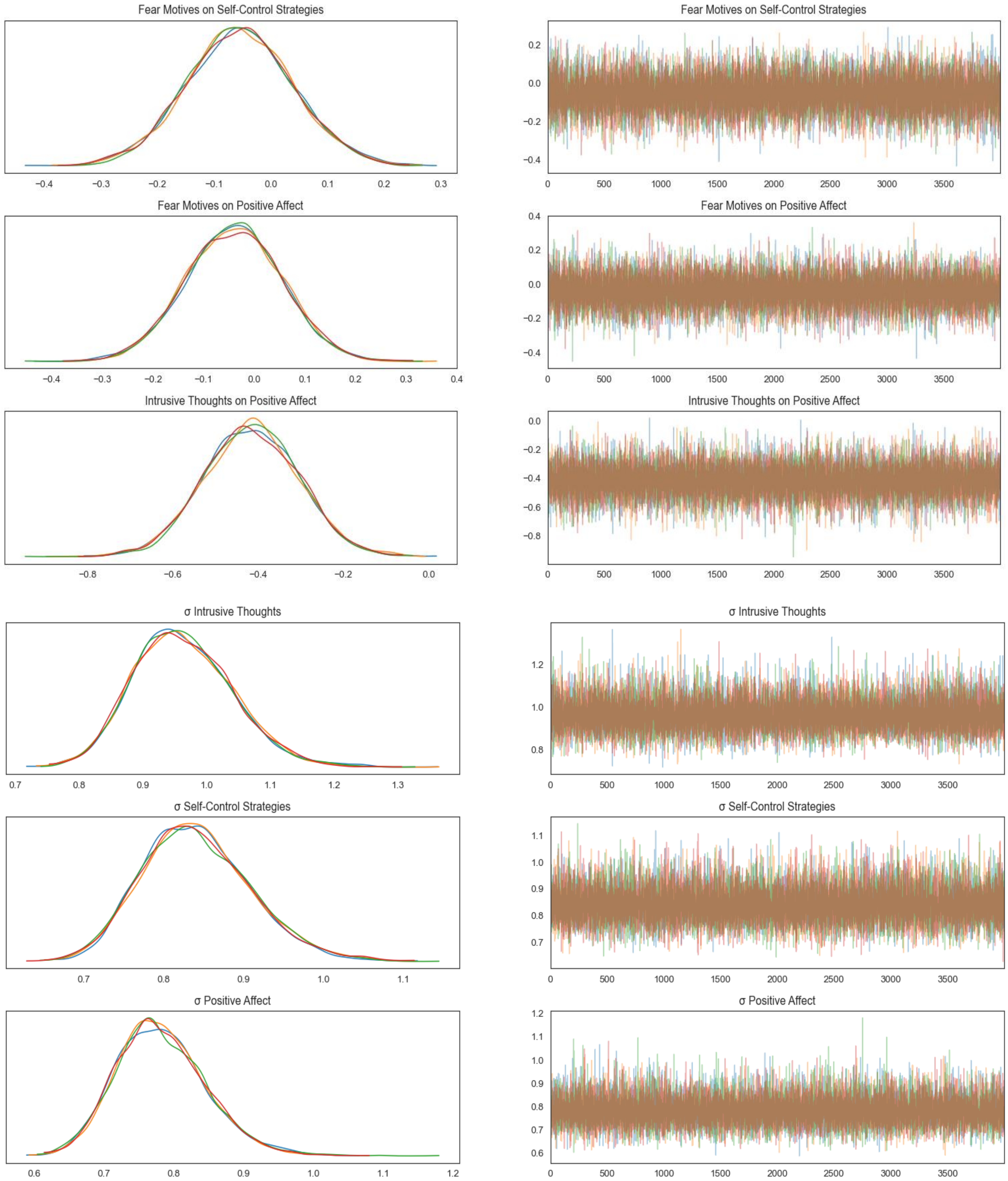
Fear Motives on Self-Control Strategies
−0.4 −0.3 −0.2 −0.1 0.0 0.1 0.2 0.3
Fear Motives on Self-Control Strategies
0.2 0.0 −0.2 −0.4
0 500 1000 1500 2000 2500 3000 3500
Fear Motives on Positive Affect
−0.4 −0.3 −0.2 −0.1 0.0 0.1 0.2 0.3 0.4
Fear Motives on Positive Affect
0.4 0.2 0.0 −0.2 −0.4
0 500 1000 1500 2000 2500 3000 3500
Intrusive Thoughts on Positive Affect
−0.8 −0.6 −0.4 −0.2 0.0
Intrusive Thoughts on Positive Affect
0.0 −0.2 −0.4 −0.6 −0.8
0 500 1000 1500 2000 2500 3000 3500
σ Intrusive Thoughts
0.7 0.8 0.9 1.0 1.1 1.2 1.3
σ Intrusive Thoughts
1.2 1.0 0.8
0 500 1000 1500 2000 2500 3000 3500
σ Self-Control Strategies
0.7 0.8 0.9 1.0 1.1
σ Self-Control Strategies
1.1 1.0 0.9 0.8 0.7
0 500 1000 1500 2000 2500 3000 3500
σ Positive Affect
0.6 0.7 0.8 0.9 1.0 1.1 1.2
σ Positive Affect
1.2 1.1 1.0 0.9 0.8 0.7 0.6
0 500 1000 1500 2000 2500 3000 3500

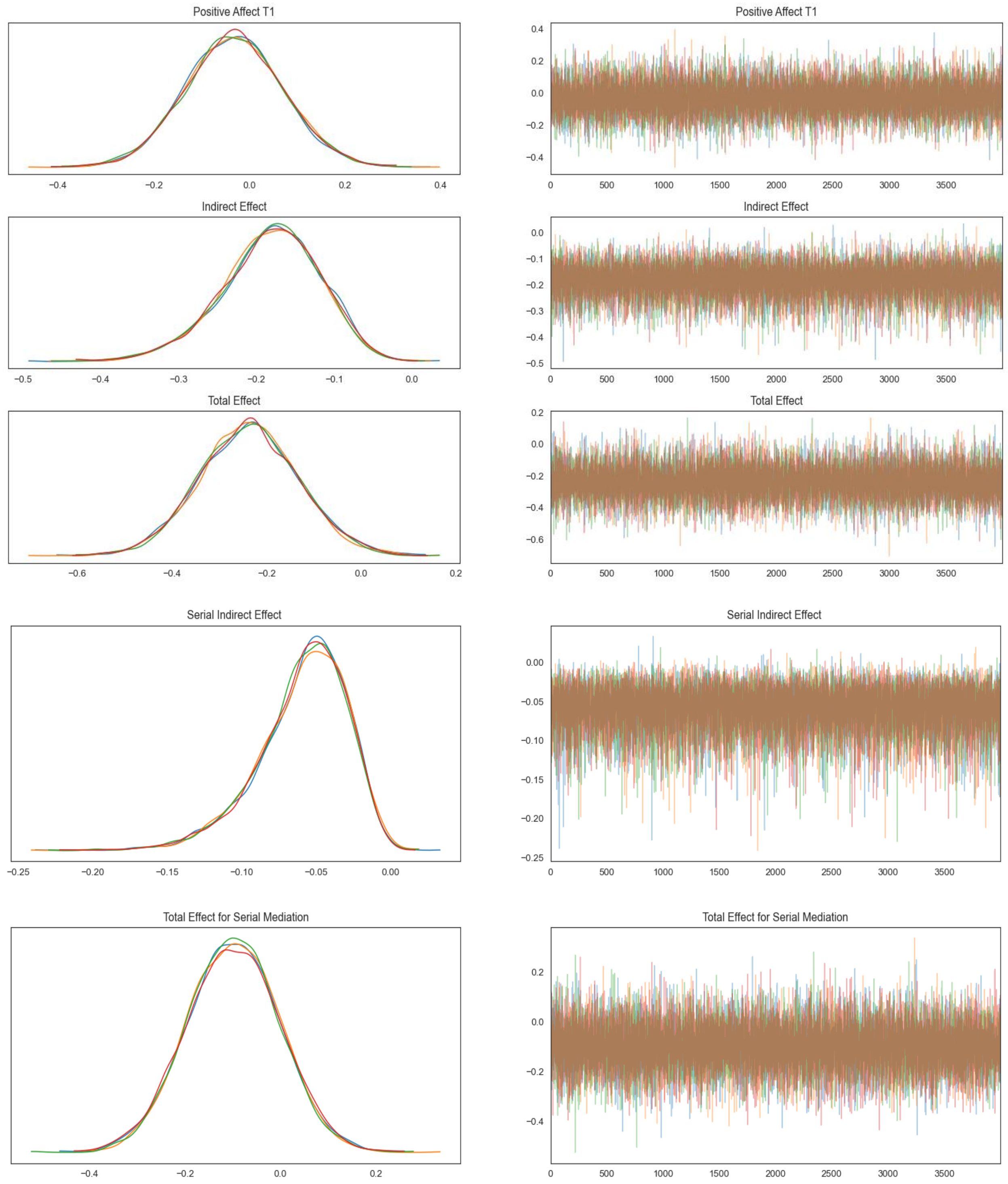

Figure S2. Trace plots for the Bayesian mediation model testing the indirect effect of fear motives on self-control strategies through intrusive thoughts and for the indirect effect of fear motives on positive affect through intrusive thoughts and self-control strategies in Study 2.

Python Code for Analyses

```
# import libraries
import pandas as pd
import numpy as np
import pymc as pm
import arviz as az
import seaborn as sns
import warnings
import matplotlib
matplotlib.use('Qt5Agg')


# set the settings
warnings.filterwarnings("ignore")
sns.set_style('white')
az.rcParams["stats.hdi_prob"] = 0.95  # set credible interval for entire notebook
az.rcParams["stats.information_criterion"] = "waic"  # set information criterion to use in
`compare`
az.rcParams["stats.ic_scale"] = "deviance"  # set information criterion scale
np.random.seed(0)


# not multprocessing but sequential
import os
# one of
os.environ['MKL_THREADING_LAYER'] = 'sequential'

# load the data
s1 = pd.read_spss('Vars_S1.sav')
s2 = pd.read_spss('Vars_S2.sav')

# Study 1 with Bayesian Mediation. Here we do not have any controls
def mediation_model(x, m, y):
    with pm.Model() as model_indirect:
        x = pm.ConstantData("x", x, dims="obs_id")
        y = pm.ConstantData("y", y, dims="obs_id")
        m = pm.ConstantData("m", m, dims="obs_id")

        # intercept priors
        im = pm.Normal("Intercept Mediator", mu=0, sigma=1)
        iy = pm.Normal("Intercept Outcome Variable", mu=0, sigma=1)

        # slope priors
        a = pm.Normal("Fear Motives", mu=0, sigma=1)
        b = pm.Normal("Intrusive Thoughts", mu=0, sigma=1)
```

```
        cprime = pm.Normal("Direct Effect", mu=0, sigma=1)

        # noise priors
        σm = pm.HalfCauchy("σ Mediator", 1)
        σy = pm.HalfCauchy("σ Outcome Variable", 1)

        # likelihood
        pm.Normal("m_likelihood", mu=im + a * x, sigma=σm, observed=m, dims="obs_id")
        pm.Normal("y_likelihood", mu=iy + b * m + cprime * x, sigma=σy, observed=y,
dims="obs_id")

        # calculate quantities of interest
        indirect_effect = pm.Deterministic("Indirect Effect", a * b)
        total_effect = pm.Deterministic("Total Effect", a * b + cprime)
    return model_indirect

# fit the model
model = mediation_model(x = s1.Fear_SUM_S, m=s1.Intrusion_Mean_S,
y=s1.SelfControl_Mean_S)

# run the posterior draws on the model
with model:
    result1 = pm.sample(draws=4000, tune=4000,
                        random_seed=42, chains=4, cores=1)

# plot trace plots
# the first three
az.plot_trace(result1, var_names=['Intercept Mediator', 'Intercept Outcome Variable',
                                  'Fear Motives'], compact=False)

# the next three
az.plot_trace(result1, var_names=['Intrusive Thoughts', "\u03c3 Mediator",
                                  "\u03c3 Outcome Variable"], compact=False)

# the last three
az.plot_trace(result1, var_names=['Direct Effect', 'Indirect Effect',
                                  'Total Effect'], compact=False)

# write the summary on a word document
summary = pm.summary(result1, round_to=2)
# write the summary to a word document
from docx import Document

# open an existing document
doc = Document()
```

```
# add a table to the end and create a reference variable
t = doc.add_table(summary.shape[0]+1, summary.shape[1])

# add the header rows.
for j in range(summary.shape[-1]):
    t.cell(0,j).text = summary.columns[j]

# add the rest of the data frame
for i in range(summary.shape[0]):
    for j in range(summary.shape[-1]):
        t.cell(i+1,j).text = str(summary.values[i,j])

# add a column with variable names
t2 = doc.add_table(summary.shape[0]+1, 1)

# add the header rows.
for j,x in enumerate(summary.index):
    t2.cell(j,0).text = summary.index[j]

# save the doc
doc.save('Summary_S1.docx')

# plot the direct, indirect and total effect
az.plot_posterior(
    result1,
    var_names=['Fear Motives', "Intrusive Thoughts", 'Indirect Effect', 'Direct Effect', "Total
Effect"],
    ref_val=0,
    hdi_prob=0.95,
    figsize=(14, 4))

# Study 2
def mediation_model(x, m1,m2, y, c1):
    with pm.Model() as model:
        x = pm.ConstantData("x", x, dims="obs_id")
        y = pm.ConstantData("y", y, dims="obs_id")
        m1 = pm.ConstantData("m1", m1, dims="obs_id")
        m2 = pm.ConstantData("m2", m2, dims="obs_id")
        c1 = pm.ConstantData("c1", c1, dims="obs_id")

        # intercept priors
        im = pm.Normal("Intercept Intrusive Thoughts", mu=0, sigma=0.27)
        im2 = pm.Normal("Intercept Self-Control Strategies", mu=0, sigma=0.24)
        iy = pm.Normal("Intercept Positive Affect", mu=0, sigma=1)
        # slope priors
        a = pm.Normal("Fear Motives", mu=0.40, sigma=0.27)
```

```
        b = pm.Normal("Intrusive Thoughts", mu=-0.58, sigma=0.27)
        c = pm.Normal("Self-Control Strategies", mu=0, sigma=1)
        control1 = pm.Normal("Positive Affect T1", mu=0, sigma=1)
        cprime = pm.Normal("Fear Motives on Self-Control Strategies", mu=-0.06, sigma=0.27)
        cprime2 = pm.Normal("Fear Motives on Positive Affect", mu=0, sigma=1)
        bprime = pm.Normal("Intrusive Thoughts on Positive Affect", mu=0, sigma=1)

        # noise priors
        σm1 = pm.HalfCauchy("σ Intrusive Thoughts", 1)
        σm2 = pm.HalfCauchy("σ Self-Control Strategies", 1)
        σy = pm.HalfCauchy("σ Positive Affect", 1)

        # likelihood
        pm.Normal("m1_likelihood", mu=im + a * x, sigma=σm1, observed=m1, dims="obs_id")
        pm.Normal("m2_likelihood", mu=im2 + b * m1 + cprime * x, sigma=σm2, observed=m2,
dims="obs_id")
        pm.Normal("y_likelihood", mu=iy + c*m2 + cprime2 * x + bprime*m1 + control1 * c1,
sigma=σy, observed=y, dims="obs_id")

        # calculate quantities of interest
        indirect_effect1 = pm.Deterministic("Indirect Effect", a * b)
        indirect_effect2 = pm.Deterministic("Serial Indirect Effect ", a * b*c)
        total_effect = pm.Deterministic("Total Effect", a * b + cprime)
        total_effect = pm.Deterministic("Total Effect for Serial Mediation", a * b * c + cprime2)
    return model

# fit the model
model = mediation_model(x=s2.Fear_SUM_S, m1=s2.Intrusion_Mean_S,
m2=s2.SelfControl_Mean_S,
                        y=s2.Pos_Aff_T2_S,c1=s2.Pos_Aff_T1_S)

# run the posterior draws on the model
with model:
    result2 = pm.sample(draws=4000, tune=4000, random_seed= 42,
                        chains=4, cores=1)

# plot trace
az.plot_trace(result2, var_names=['Intercept Intrusive Thoughts', 'Intercept Self-Control
Strategies',
                                  'Intercept Positive Affect'], compact=False)
az.plot_trace(result2, var_names=['Fear Motives', 'Intrusive Thoughts',
                                  'Self-Control Strategies'], compact=False)
az.plot_trace(result2, var_names=['Fear Motives on Self-Control Strategies',
                                  'Fear Motives on Positive Affect',
                                  'Intrusive Thoughts on Positive Affect'], compact=False)
az.plot_trace(result2, var_names=['\u03c3 Intrusive Thoughts', '\u03c3 Self-Control Strategies',
```

```
                                  '\u03c3 Positive Affect'], compact=False)
az.plot_trace(result2, var_names=['Positive Affect T1', 'Indirect Effect',
                                  'Total Effect'], compact=False)
az.plot_trace(result2, var_names=['Serial Indirect Effect ',
                                  'Total Effect for Serial Mediation'], compact=False)

# plot the 95% HDI for only the variables we are interested in
az.plot_posterior(
    result2,
    var_names=['Intrusive Thoughts', 'Self-Control Strategies',
               "Indirect Effect", "Total Effect",
               "Serial Indirect Effect ", "Total Effect for Serial Mediation"],
    ref_val=0,
    hdi_prob=0.95)

# print the results
summary2 = pm.summary(result2, round_to=2)

# write the summary to a word document
import docx

# open an existing document
doc = docx.Document()

# add a table to the end and create a reference variable
t = doc.add_table(summary2.shape[0]+1, summary2.shape[1])

# add the header rows.
for j in range(summary2.shape[-1]):
    t.cell(0,j).text = summary2.columns[j]

# add the rest of the data frame
for i in range(summary2.shape[0]):
    for j in range(summary2.shape[-1]):
        t.cell(i+1,j).text = str(summary2.values[i,j])

# add a column with variable names
t2 = doc.add_table(summary2.shape[0]+1, 1)

# add the header rows.
for j,x in enumerate(summary2.index):
    t2.cell(j,0).text = summary2.index[j]
# save the doc
doc.save('Summary_S2.docx')
```
